# Optimization and standardization of transient expression assays for gene functional analyses in strawberry fruits

**DOI:** 10.1038/s41438-019-0135-5

**Published:** 2019-05-01

**Authors:** Yaoyao Zhao, Wenwen Mao, Yating Chen, Wei Wang, Zhengrong Dai, Zhechao Dou, Kai Zhang, Lingzhi Wei, Tianyu Li, Baozhen Zeng, Ting Liu, Yijuan Fan, Jiaqi Yan, Bingbing Li, Wensuo Jia

**Affiliations:** College of Horticulture, China Agriculture University, Beijing, CN 100193 P.R. China

**Keywords:** Plant sciences, Plant biotechnology, Plant sciences, Plant biotechnology

## Abstract

Strawberry is increasingly used as a model plant for research on fruit growth and development. The transient gene manipulation (TGM) technique is widely used to determine the function of plant genes, including those in strawberry fruits. However, its reliable application for the precise identification of gene function has been difficult owing to the lack of conditional optimization. In this study, we found that successful transient gene manipulation requires optimization, with the vector type, temperature, and fruit developmental stage being three major factors determining success. Notably, we found that transient gene manipulation was feasible only from the large green fruit stage onwards, making it especially suitable for identifying genes involved in strawberry fruit ripening. Furthermore, we established a method called percentage difference of phenotype (PDP), in which the functional effect of a gene could be precisely and efficiently identified in strawberry fruits. This method can be used to estimate the functional effect of a gene as a value from 0 to 100%, such that different genes can be quantitatively compared for their relative abilities to regulate fruit ripening. This study provides a useful tool for accelerating research on the molecular basis of strawberry fruit ripening.

## Crop genetics: genetic tools to study strawberry ripening

Chinese researchers have optimized transient gene manipulation (TGM) to probe the function of genes in strawberries. In TGM, the expression of a gene is temporarily altered and the effect on plant development measured. TGM experiments can be conducted in days rather than the months needed for experiments involving stable transformation. A team led by Jia Wensuo of China Agriculture University evaluated how the use of TGM in strawberry fruits was affected by various conditions, including different delivery vectors, temperatures, time periods, and fruit stages. They found that the correct delivery vector, temperature, and fruit stage were crucial for the success of TGM. The team also developed a TGM analysis method to quantify and compare the contribution of different genes. The techniques developed in this study provide valuable tools for the genetic analysis of strawberry ripening.

## Introduction

Fleshy fruits are physiologically classified as either climacteric or nonclimacteric. Research into the molecular basis of fleshy fruit growth and development has increasingly attracted attention from agricultural and plant scientists^1–5^. Whereas extensive research has been carried out in tomato (*Solanum lycopersicum*), the model climacteric fruit species, little work has focused on nonclimacteric fruits, largely due to the lack of a suitable research system (in terms of both materials and methods). Strawberry is emerging as a model nonclimacteric fruit plant^2,6^. However, the gene expression manipulation crucial to molecular research is much more challenging in strawberry than in tomato. For example, stable gene transformation of strawberry is time-consuming, requiring at least a year and a half from initial explant transformation to first fruit ripening^7^, and this is a major factor limiting the progress of molecular research in nonclimacteric fruits.

Transient gene manipulation (TGM) is a powerful technique that can identify gene function in a matter of days and can therefore be used to screen a number of genes potentially involved in the regulation of fruit growth and ripening in a short time frame. Spolaore et al.^8^ demonstrated that strawberry fruits are amenable to transient gene expression; strawberries injected with *Agrobacterium tumefaciens* transformed with p35SGUSINT displayed β-glucuronidase (*GUS*) reporter gene activity. A study by Agius F. et al.^9^ based on the biolistic transformation method showed that transient gene expression in strawberry fruits could be used to analyze promoter activity. Ribonucleic acid interference (RNAi), which has been extensively used for gene silencing in plants, can be initiated by the delivery of double-stranded RNA mediated by *Agrobacterium* or a plant virus^10–13^. Using the chalcone synthase gene (*CHS*) as a reporter, Hoffman et al.^7^ tested the RNAi-based transient silencing method in strawberry fruits. The authors injected an *Agrobacterium* strain carrying a *CHS* “hairpin” RNA construct into the receptacles of attached fruits and showed that this caused a reduction in *CHS* mRNA abundance as well as CHS enzymatic activity. Virus-induced gene silencing (VIGS) has been used to silence genes in tomato fruits^11^. In strawberry fruits, there have been few reports of successful use of VIGS to identify gene function, and it was shown that VIGS could be used to silence *FaBG1* (*β-glucosidase 1*), and *FaNCED1* (*nine-cis-epoxycarotenoid dioxygenase*)^14–20^.

Since the successful establishment of TGM more than 10 years ago^7–9^, reports of its application in strawberry fruit molecular research have been limited. We recently attempted to use TGM for functional gene studies in strawberry fruits^21–23^ and found that this approach was far more challenging than expected. Indeed, TGM may even yield incorrect conclusions if errors are introduced. Three main factors contribute to this complexity. (1) Gene functional identification is based mainly on the observation of phenotypic changes caused by gene manipulation (i.e., the phenotypic difference between treatment and control groups). For studies of strawberry fruit ripening, observing changes in fruit pigment accumulation is the simplest strategy. However, even if a target gene could be effectively manipulated, such a manipulation might not trigger a dramatic change in pigment accumulation because the regulatory time of the gene’s expression might be too short. Alternatively, even if manipulating a gene may potentially induce a phenotypic change, that change may be masked by an innate difference between treatment and control fruits (since for any two fruits, the developmental process might not be absolutely identical), and this could result in failure to identify the gene’s function or in an incorrect conclusion. (2) As many factors may influence the effectiveness of TGM, an improperly conducted TGM may not effectively regulate the target gene’s expression. (3) For studies of gene function, we may be interested not only in identifying the functions of specific genes but also in comparing a given set of genes for their relative abilities to regulate a developmental process. We therefore designed this study to investigate the factors that affect TGM, with the aim of optimizing TGM conditions in strawberry to maximally manipulate gene expression. Furthermore, we developed a method, named percentage difference of phenotype (PDP), to effectively and precisely determine whether a gene is a regulator of strawberry fruit ripening.

## Materials and methods

### Plant materials and growth conditions

Octoploid strawberry plants (*Fragaria* × *ananassa* Duch., Benihoppe) were grown in a greenhouse at 18–28 °C and 75–90% humidity under an 8-h/16-h dark/light cycle. The fruits were classified into six developmental stages as follows: small green fruit (SG), mid-sized green fruit (MG), large green fruit (LG), white fruit (W), turning fruit (T), and fully reddened fruit (FR)^21–23^. Either attached or detached fruits at different stages were used depending on the goals of the experiment.

### Vector construction

The Gateway vector PKGWD (*p35S*::eGFP-*pACP1*/*pEXP2*/*p35S*::GUS), carrying the *eGFP* reporter gene driven by the cauliflower mosaic virus 35 S promoter, was donated by Kevin Folta’s laboratory (University of Florida) and used to express the marker genes *eGFP* and *GUS*. We previously screened *pEXP2* (the promoter of *EXPANSINS2* [*EXP2*]) and *pACP1* (the promoter of *ACYL CARRIER PROTEIN1* [*ACP1*]) for octoploid and diploid strawberry fruit-specific expression promoters, which show strong driving power in strawberry fruits^24,25^. *eGFP* was driven by the 35 S promoter, and *GUS* was driven variously by *pACP1*, *pEXP2*, or the 35S promoter. Virus-induced gene silencing (VIGS) in strawberry fruits was carried out with the plasmid vectors pTRV1 and pTRV2^11^. To construct the overexpression vector FaMYB10^26^, the full-length coding sequence of FaMYB10 was amplified with the sense primer 5′-ATGGAGGGTTTCGGTGTGAGAAAAG-3′ and antisense primer 5′-TCATACGTAGGAGATGTTGACTAGATC-3′, and then the PCR products were introduced into pBI121 with *XbaI* and *BamHI*, such that their expression was driven by the 35S promoter.

### Fruit TGM

*Agrobacterium tumefaciens* strain EHA105 was used to perform transient expression analyses in strawberry fruits. *Agrobacterium* was grown at 28 °C in LB liquid medium with appropriate antibiotics. When the culture reached an OD_600_ of approximately 0.8, the cells were resuspended in infection buffer (10 mM MgCl_2_, 100 μM acetosyringone, and 10 mM MES, pH 5.6) and shaken for 1 h at room temperature before being used. For *Agrobacterium* infection, the *Agrobacterium* suspension was injected into the fruit using a syringe of 1 mL capacity. To do this, the needle tip was inserted into the fruit center from the top, and then the *Agrobacterium* suspension was slowly and evenly injected into the fruits until the strawberry fruit was completely infected. After the infection, the fruits were incubated under the conditions required for the different experimental aims. The effect of TGM was evaluated by examining the changes in both reporter gene expression and protein accumulation at different time points after *Agrobacterium* infection.

### Quantitative reverse transcription PCR (RT-qPCR)

Strawberry fruits were ground to a powder in liquid nitrogen, and then total RNA was isolated using the procedure of the E.Z.N.A. Total RNA Kit (OMEGA). One microgram of total RNA was used to synthesize the cDNA with M-MLV Reverse Transcriptase (Promega) according to the manufacturer’s instructions. RT-qPCR was performed using SYBR Premix Ex Taq (TaKaRa) in an ABI7500 Real-Time PCR System (Applied Biosystems). RT-qPCR primers were designed using Primer3 Plus^3^. Each sample was analyzed in triplicate. *FaACTIN* was used as an internal control, and the 2^-△△CT^ method was used to determine transcription levels.

### Immunoblot analysis

Strawberry fruit proteins were extracted from detached strawberry fruits that had been agroinfiltrated with the Gateway vector PKGWD (*p35S*::eGFP). 4 days after transfection, the fruits were ground in liquid nitrogen to a fine powder and transferred into 1 mL of cold extraction buffer (1 mM EDTA, 10% glycerin, 0.5% Triton X-100, 1 mM DTT, and 100 mM Na_2_HPO_4_-NaH_2_PO_4_), briefly mixed with a vortex mixer, and then incubated on ice for 20 min. The protein extracts were centrifuged at 4 °C, 13,000×*g*, for 10 min, and the supernatant was collected into a new EP tube. SDS loading buffer was added, and the mixture was boiled for 5 min. Protein samples were fractionated by SDS-PAGE electrophoresis, and immunoblot analysis was performed using an anti-eGFP monoclonal antibody (cwbio, CW0295S).

### eGFP imaging analysis

For eGFP fluorescence imaging, we developed an apparatus with an argon laser, a 488-nm excitation filter, and a 507-nm emission filter for analyzing and imaging objects up to 100 cm^2^ in surface area, suitable for assays of common fruits.

### Conditional optimization of fruit transient gene expression

(1) Pattern of gene expression and protein accumulation among individual fruits

Thirty detached fruits were individually infected with *Agrobacterium* carrying the *eGFP* reporter gene as described above. After injection, the fruits were incubated at 23(±2) °C and 100% humidity. Four days after infection, eGFP protein and gene expression were analyzed by fluorescence imaging and RT-qPCR, respectively.

(2) Effect of promoter on TGM

To examine the effect of the promoter on TGM, we used the three PKGWD Gateway vectors described above, carrying the reporter genes *eGFP* (driven by *p35S*) and *GUS* (driven by *p35S*, *pACP1*, or *pEXP2*, respectively). For GUS activity analysis, the fruit sample was incubated in staining buffer [10 mL X-Gluc, 1 mL K_4_Fe(CN)_6_·3H_2_O (50 mM), 1 mL K_3_Fe(CN)_6_ (50 mM), 10 mL CH_3_OH, 1 mL 10% Triton X-100, and 77 mL phosphate buffer] for 8 h. After incubation, the fruit was decolored with alcohol to observe the activity of the GUS protein. For RT-qPCR analysis, three individual fruits were mixed as a sample.

(3) Effects of *Agrobacterium* quantity and fruit attachment status on TGM

This experiment included two parts: investigating the effectiveness of TGM in relation to fruit status, i.e., comparing its effects in detached and attached fruits, and in relation to the amount of *Agrobacterium* delivered to the fruit. For TGM with attached fruits, fruits at the LG stage were used. After *Agrobacterium* infection, fruits were grown normally under the conditions described above, and 4 days after infection, eGFP protein and gene expression were analyzed by fluorescence imaging and RT-qPCR, respectively. For TGM with detached fruits, after *Agrobacterium* infection, fruits were incubated at 23(±2) °C and 100% humidity. Four days after the infection, eGFP protein and gene expression were analyzed by fluorescence imaging and RT-qPCR, respectively. To study TGM in relation to the delivered amount of *Agrobacterium*, detached LG fruits were injected with *Agrobacterium* in one of two ways: either full injection, in which each individual fruit was injected with *Agrobacterium* until the whole fruit was fully infiltrated, or quantitative injection, in which each fruit was injected with 0.5 mL of *Agrobacterium*. Four days after the infection, eGFP protein and gene expression were analyzed by fluorescence imaging and RT-qPCR, respectively.

(4) Effect of temperature on TGM

Detached LG fruits were infected with *Agrobacterium* by full injection, as described above. After the infection, the fruits were incubated at different temperatures as follows: 15, 20, 25, 30, and 35 °C. Four days after the infection, eGFP protein and gene expression were analyzed by fluorescence imaging and RT-qPCR, respectively.

(5) Effect of time on TGM

Detached LG fruits were infected with *Agrobacterium* by full injection as described above. After injection, the fruits were incubated at 23(±2) °C and 100% humidity. To evaluate the changes in the level of eGFP protein, immunoblot analysis was conducted, in addition to fluorescence imaging, as described above. The eGFP protein imaging, immunoblotting, and gene expression analysis were conducted at 0, 2, 4, 6, 8, and 10 days after infection.

(6) Effect of fruit developmental stage on TGM

The detached fruits were picked at the SG, MG, LG, and W stages, and TGM was conducted as described above. Four days after *Agrobacterium* infection, eGFP protein imaging, immunoblotting, and gene expression analysis were conducted as described above.

### PDP analysis

The PDP analysis was essentially based on a statistical analysis of the difference in the phenotypic change in paired fruits that were transformed with either a target gene or an empty vector (the control). To do this, a batch of fruits was detached and immediately taken to the laboratory. To avoid water loss, once detached, the fruits were maintained at 100% humidity. Fruits were paired based on their developmental stage. Assessment of the fruit developmental stage is based on comprehensive parameters such as color, size, shape, and swollen status of the receptacle. Then, one of the paired fruits was transformed with a target gene and the other with the empty vector control. After vector transformation, pigment accumulation was continuously examined until one of the paired fruits started to redden. The fruit that started to redden first was marked “1,” and the other fruit was marked “0.” If both paired fruits became red at the same time, they were both marked “1.” After pigment accumulation had been examined, the percentage of fruits marked “1” relative to the total number of fruit pairs was calculated. The percentage difference between the fruits transformed with a target gene and the fruits transformed with the control vector was designated as the PDP value, which varies from 0 to 100%.

## Results

### Pattern of gene expression and protein accumulation among individual fruits

**Fig. 1:**
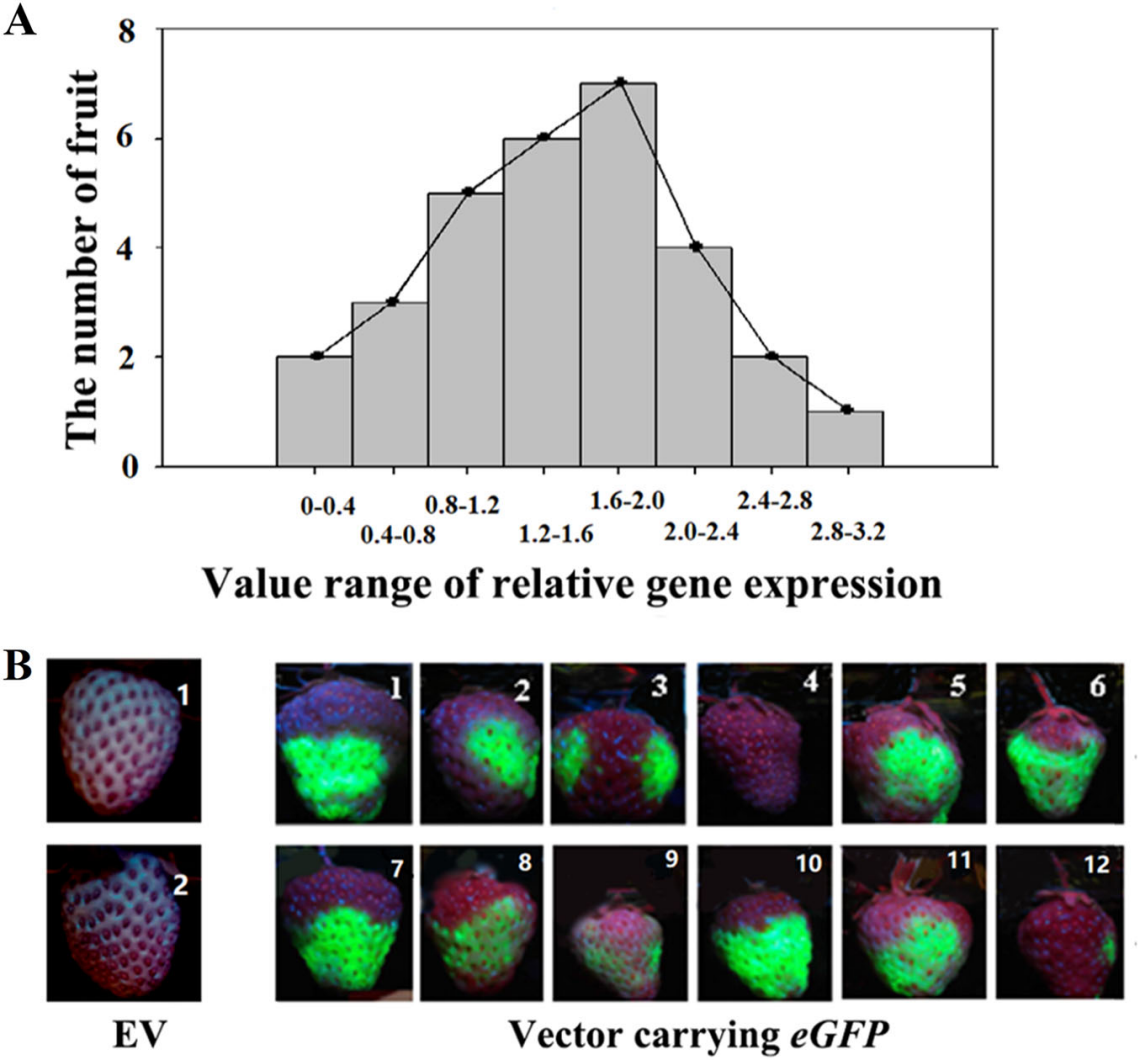
Pattern of *eGFP* expression and protein accumulation among individual fruits. **a** RT-qPCR analysis of *eGFP* expression, showing variation in characteristics among individual fruits. Detached fruits with a specimen number of 30 were individually infected with *Agrobacterium* carrying the *eGFP* reporter gene, and gene expression was assessed 4 days after infection. The horizontal ordinate represents relative gene expression, and the whole value range was divided into successive subranges with a span of 0.4. The vertical ordinate represents the number of fruits with the values of gene expression within each subrange. *FaACTIN* was used as a normalized control gene. **b** eGFP protein images showing a variation in pattern of eGFP protein accumulation. Twelve representative fruits are shown. *EV* empty vector

To demonstrate whether TGM could be well established, we first examined whether an *eGFP* reporter gene could be highly expressed and whether the expression level was basically identical among individual fruits. Unexpectedly, we found that the pattern of *eGFP* expression varied greatly among individual fruits; i.e., while expression was high in some fruits, it was low or even absent in others. To characterize the variation pattern, we compared the relative levels of *eGFP* expression among 30 individual fruits. While the sample showed a basically normal distribution of relative expression levels, the relative gene expression values varied greatly among individual fruits, as reflected by the related statistical parameters (e.g., the large range from 0 to 3.0 and the large ratio of confidence/mean, i.e., a 95% confidence of 0.6978 versus a mean of 1.5114; Fig. 1a). Consistent with the analysis of gene expression, imaging analysis also showed a large variation in eGFP accumulation among different individual fruits (Fig. 1b); while strong fluorescence was observed in some fruits, much weaker or even no fluorescence was found in others.

### Effect of gene promoter on TGM

**Fig. 2:**
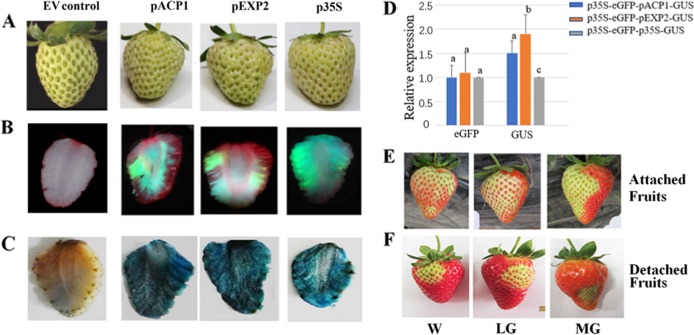
Effect of vector type on transient gene manipulation. **a** Detached fruits ready for infection, showing phenotypic identity among the fruits. Three different vectors were compared. All three vectors contained eGFP driven by the p35S promoter and GUS driven by the pACP1, pEXP2, or p35S CaMV promoters. **b** eGFP fluorescence images on the 5th day after *Agrobacterium* infection. Three representative fruits are shown. **c** GUS staining on the 5th day after *Agrobacterium* infection. Three representative fruits are shown. **d** qRT-PCR analysis of eGFP and GUS expression on the 5th day after *Agrobacterium* infection. *FaACTIN* as a normalized control gene. EV, empty vector. Values are the mean ± SD of six fruits. Statistically significant differences among samples were tested by Tukey’s test, and significant differences at the *P* < 0.05 level are indicated by different letters. E and F. Attached (**e**) and detached fruits (**f**) were infected with a VIGS empty vector at three different stages, and photographs were taken when the fruits started to turn red

As the transcriptional activity of a gene is determined by the promoter driving its expression, we examined the effect of different promoters on TGM using *eGFP* and *GUS* as reporter genes. We constructed three different vectors carrying both *eGFP* and *GUS* reporter genes; all three had *eGFP* expression driven by the *p35S* promoter, but *GUS* expression was driven by one of three promoters, *pACP1*, *pEXP2*, or *p35S*. The three vectors were transformed into strawberry fruits with identical phenotypes at the LG stage (Fig. 2a). As shown in Fig. 2b, among the three vectors, we found little difference in eGFP levels upon imaging, but significant differences in GUS staining (Fig. 2c), with *pEXP2* showing the strongest driving activity among the three promoters. Consistent with these observations, RT-qPCR analysis clearly showed that, whereas the *eGFP* gene transcript levels were basically the same for all three vectors (Fig. 2d), the *GUS* transcript levels showed differences, with the highest and lowest levels associated with *pEXP2* and *p35S*, respectively (Fig. 2d). These observations indicate that the promoter is an important factor affecting TGM.

VIGS is a powerful tool for genetic studies in some plants and plant organs, such as tomato fruits^11^. Therefore, in this study, we also examined factors that might affect the application of VIGS to strawberry fruits. To this end, we examined the effect of VIGS-mediated silencing of *MYB10*, which encodes a key protein controlling pigment accumulation. Since VIGS could potentially damage fruit cells, we first evaluated whether the VIGS vector itself might affect the fruit phenotype by injecting the empty vector into attached (Fig. 2e) or detached (Fig. 2f) fruits at the MG, LG, or W stages. Unexpectedly, this caused serious damage to the fruit, as indicated by a strong inhibition of pigment accumulation. Because of this, as well as the poor reproducibility problems we and others^27,28^ observed, we did not pursue VIGS analyses further in strawberry fruits.

### Effect of *Agrobacterium* delivery protocol on TGM

**Fig. 3:**
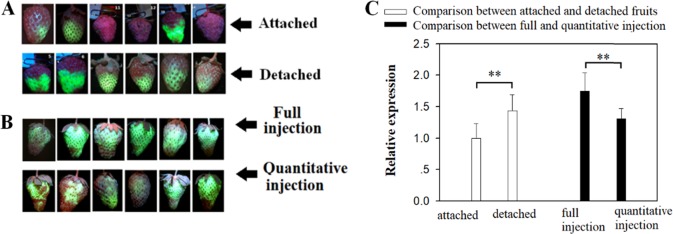
Effect of *Agrobacterium* infection method on transient gene manipulation. **a** Comparison between attached and detached fruits injected with *Agrobacterium* carrying the *eGFP* reporter gene driven by the 35CaMV promoter. Six individual fruits are shown to demonstrate a variation in eGFP images. **b**. Comparison between two different methods of *Agrobacterium* infection. For full injection, the *Agrobacterium* suspension was evenly injected into the fruits until fully infiltrated, and for defined (quantitative) injection, each individual fruit was injected with 0.5 mL of *Agrobacterium* suspension. Six individual fruits are shown to demonstrate a variation in eGFP images. **c**. qRT-PCR analysis of *eGFP* expression for comparisons of different methods of transient gene manipulation. The analysis was conducted on the 5th day after *Agrobacterium* infection. *FaACTIN* was used as a normalized control gene. Values are means ± SD of six fruits. *P* values were calculated by Student’s *t* test: *P < 0.05

In using TGM to study gene function, there were two factors that deserved particular attention: (1) whether to use attached or detached fruits and (2) whether to deliver a defined amount (quantitative injection) or a maximum amount of *Agrobacterium* (full injection) to each fruit. Using attached fruits resulted in less damage, but using detached fruits facilitated their manipulation. Furthermore, using a defined amount of *Agrobacterium* would be expected to result in better uniformity of treatment between fruits, which was especially important for a comparison between treated and control fruits, but using a maximum amount of *Agrobacterium* was expected to be better in regard to its ability to manipulate gene expression. Given these concerns, we studied TGM in relation to the fruit type (i.e., attached versus detached) and the amount of *Agrobacterium* (i.e., a defined amount versus a maximum amount). To this end, we injected a suspension of *Agrobacterium* carrying the *eGFP* reporter gene into LG fruits and then examined the fruits for *eGFP* gene expression by RT-qPCR, as well as for eGFP protein accumulation by eGFP imaging. As shown in Fig. 3, regardless of fruit type and amount of *Agrobacterium*, the eGFP protein level varied greatly among individual fruits, especially for the attached fruits. Nevertheless, the eGFP protein level was higher overall in detached than in attached fruits (Fig. 3a). Consistent with the pattern of eGFP accumulation, the expression of eGFP was also significantly higher in detached than in attached fruits (Fig. 3b). In the group receiving a defined amount of *Agrobacterium*, each individual fruit was injected with 0.5 mL of the suspension, and in the group receiving a maximum amount of *Agrobacterium*, the suspension was injected into fruits until each was fully infiltrated (designated hereafter as “full injection”). As shown in Fig. 3c, the level of eGFP protein was much higher after the full injection than after the defined 0.5-mL injection. Consistent with this, the level of eGFP expression was also significantly higher after the full injection than after the defined injection.

### Effect of temperature on TGM

**Fig. 4:**
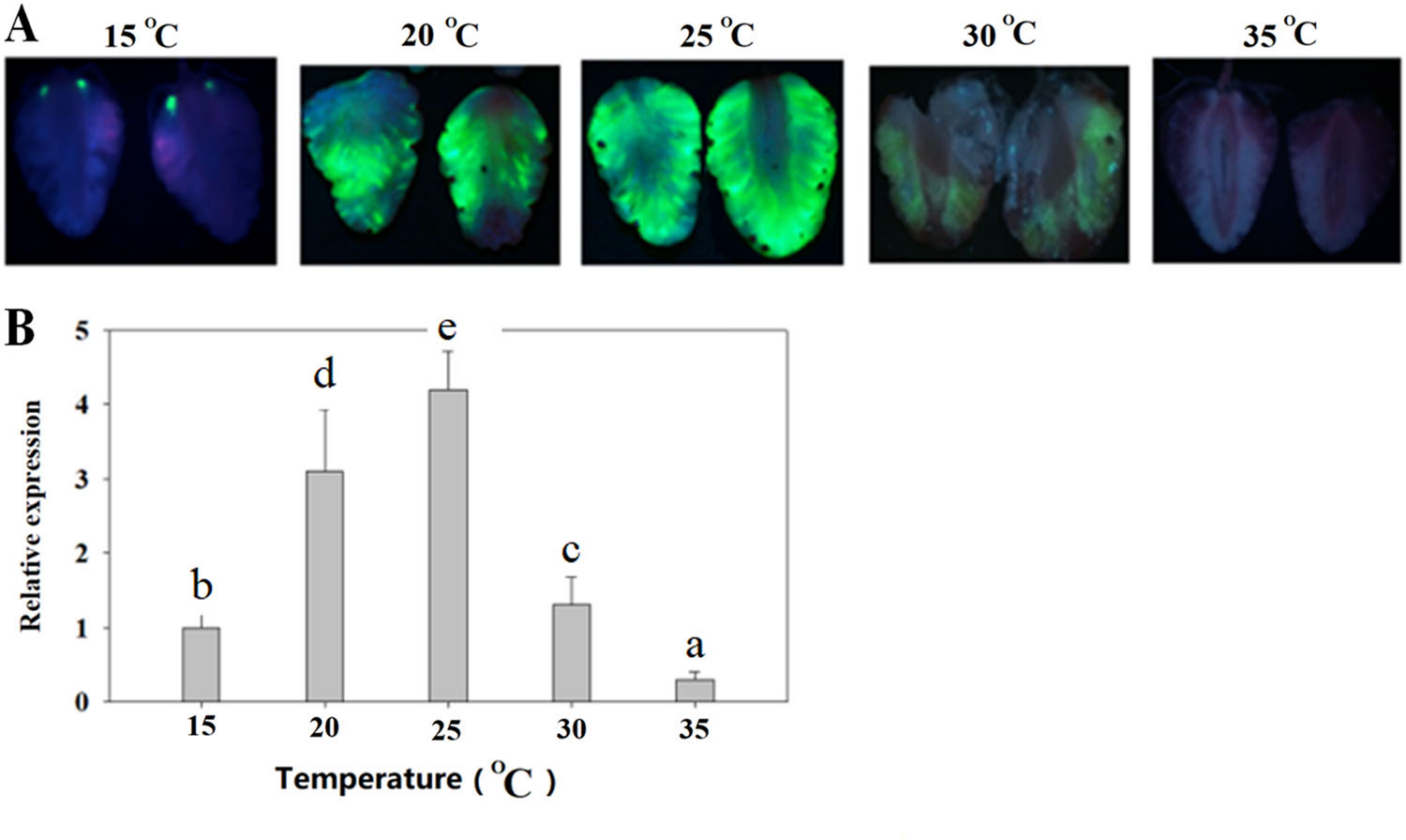
Effect of temperature on transient gene manipulation. Detached LG fruits were infected with *Agrobacterium* carrying the *eGFP* reporter gene driven by the 35CaMV promoter. After infection, fruits were incubated at different temperatures at 100% humidity. eGFP image assays were conducted on the 5th day after *Agrobacterium* infection. **a** eGFP images. **b** qRT-PCR analysis of *eGFP* gene expression. *FaACTIN* was used as a normalized control gene. Values are means ± SD of six fruits. Statistically significant differences among samples were tested by Tukey’s test, and significant differences at the *P* < 0.05 level are indicated by different letters

As environmental temperature inevitably changes, we examined whether temperature might affect TGM. To this end, we carried out full injections of detached LG fruits with *Agrobacterium* carrying the *eGFP* reporter gene and then incubated the fruits at different temperatures. Indeed, both eGFP protein accumulation and *eGFP* gene expression were strongly affected by temperature. Whereas eGFP protein accumulated strongly at temperatures of 20 to 25 °C, the western blot did not show eGFP protein accumulation at temperatures below 15 or above 30 °C (Fig. 4). Consistent with this, maximum *eGFP* expression also occurred between 20 and 25 °C. These observations suggest that temperature may be a critical factor affecting TGM.

### Effect of time on TGM

**Fig. 5:**
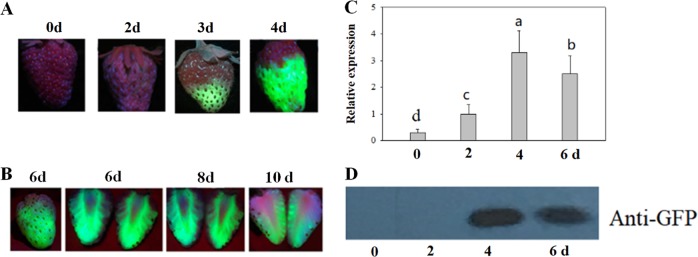
Effect of incubation time on transient gene manipulation. Detached LG fruits were infected with *Agrobacterium* carrying the *eGFP* gene driven by the 35CaMV promoter, and the fruits were incubated for different times at 25 °C and 100% humidity. **a** eGFP images on different days after *Agrobacterium* infection. **b** eGFP image of a representative fruit, showing the altered pattern a relatively long time after *Agrobacterium* infection. C. qRT-PCR analysis of eGFP gene expression. *FaACTIN* was used as a normalized control gene. Values are means ± SD of six fruits. Statistically significant differences among samples were determined by Tukey’s test, and significant differences at the *P* < 0.05 level are indicated by different letters. D. Immunoblot analysis of eGFP protein; each lane represents a mixed sample of three fruits from different plants

During fruit development and ripening, the expression patterns of a series of ripening-related genes are altered. Accordingly, when identifying gene function, the timing of target gene manipulation has a particularly important impact on the effectiveness of that manipulation. With *eGFP* as a reporter gene, we examined the changes in the patterns of eGFP protein accumulation, as well as gene expression, in the course of TGM. As shown in Fig. 5, *eGFP* started to accumulate on the 3rd day after *Agrobacterium* injection and peaked from the 4th to the 5th day (Figs. 5a, d). Surprisingly, eGFP protein accumulation can continue for more than 10 days after *Agrobacterium* injection (Fig. 5b). Figure 5c shows the time course of *eGFP* expression. Some *eGFP* transcripts were detected immediately after *Agrobacterium* injection, when no eGFP protein accumulation could be detected yet, suggesting that the *eGFP* transcript observed at this point might be derived from *Agrobacterium* cells rather than fruit cells. This result implies that, as a means to demonstrate whether a target gene may be successfully manipulated by TGM, examining target gene expression alone is actually not accurate. Rather, owing to the contribution of *Agrobacterium* itself to target gene transcript accumulation, accurately determining the pattern of TGM, especially in the case of gene overexpression, requires an examination of protein accumulation rather than of the gene transcript level.

### Effect of fruit developmental stage on TGM

**Fig. 6:**
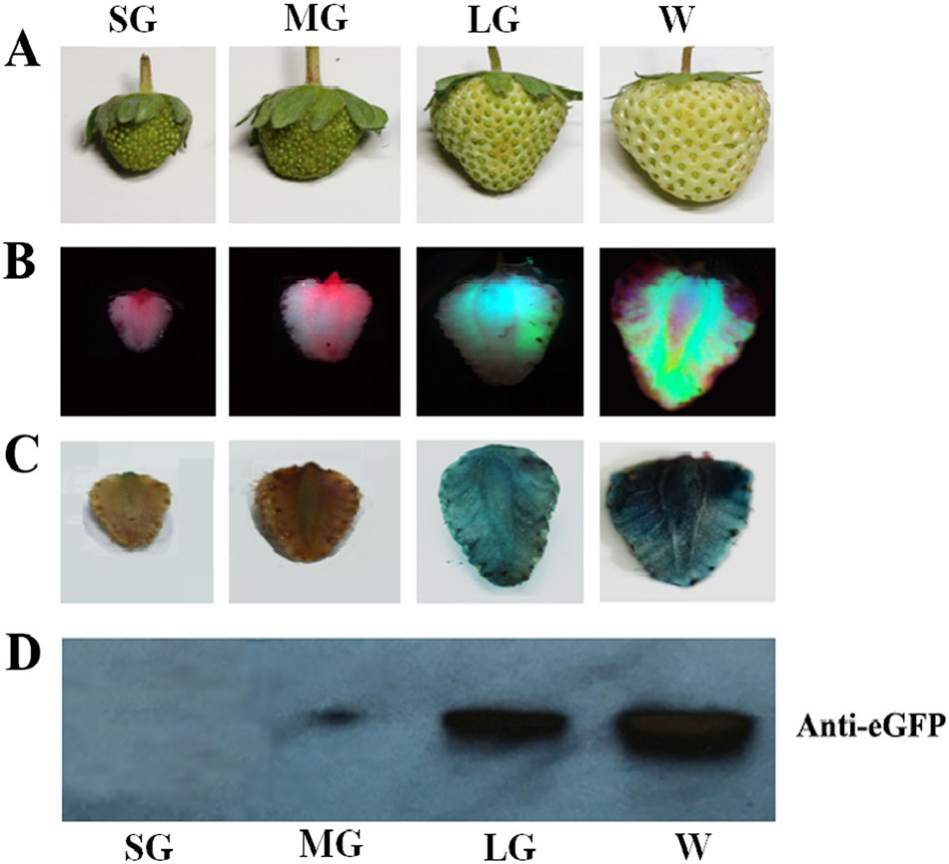
Effect of fruit developmental stage on transient gene manipulation. Detached fruits of different stages were infected by injection of *Agrobacterium* carrying the vector containing both the *eGFP* and *GUS* reporter genes driven by the 35CaMV promoter. Five days after infection, eGFP protein was imaged, and GUS activity was stained. **a** Fruits at different developmental stages: *SG* small green fruit, *MG* mid-sized green fruit, *LG* large green fruit, *W* white fruit. **b** eGFP images. **c** GUS staining. **d** Immunoblot analysis of eGFP protein; each lane represents a mixed sample of three fruits from different plants

Fruit growth and development are complex processes in which different genes play different roles at different stages. Hence, for gene functional identification, it is important to know whether TGM is suitable for the fruit developmental stage of interest. We therefore examined TGM in relation to fruit development, dividing the fruit developmental process into four major stages: the small green (SG), mid-sized green (MG), large green (LG), and white stages (W) (Fig. 6a). The reddening stage was not included because it was too late to conduct gene functional identification. In this experiment, we studied TGM with a vector carrying both *eGFP* and *GUS* driven by the *p35S* promoter. As shown in Figs. 6b, c, eGFP and GUS protein accumulation, as evidenced by both eGFP imaging and GUS staining, was detected only at the LG and W stages, and no protein was detected before the LG stage. To quantify the pattern of protein accumulation, we conducted an immunoblot analysis of eGFP. Consistent with the observations from eGFP imaging and GUS staining, the immunoblot analysis suggests that the later the developmental stage, the higher the levels of the two proteins (Fig. 6d). This result appears to imply that TGM may only be suitable for studies of fruit ripening but not for studies of other stages.

### Effect of strawberry variety on TGM

**Fig. 7:**
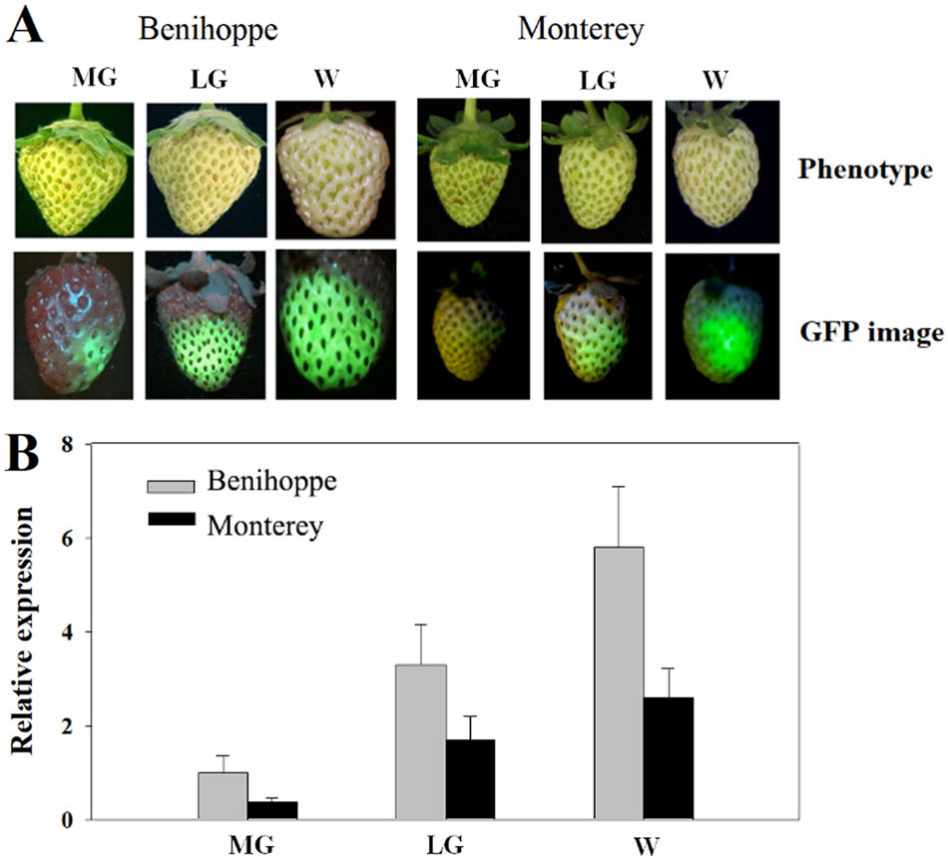
Transient gene manipulation in relation to strawberry varieties. Detached fruits at different stages were infected with *Agrobacterium* carrying the *eGFP* gene driven by the 35CaMV promoter, and the fruits were incubated for 5 days at 25 °C and 100% humidity. **a** Fruits were detached at the MG, LG, and W stages, comparison between two strawberry varieties (Benihoppe versus Monterey) with respect to phenotypes (upper panel) and GFP fluorescence (lower panel). **b** qRT-PCR analysis of *eGFP* expression. *FaACTIN* was used as a normalized control gene. Values are means ± SD of six fruits. Statistically significant differences among samples were determined by Tukey’s test, and significant differences at the *P* < 0.05 level are indicated by different letters

We tested whether TGM could be used in different strawberry varieties, including *Fragaria ananassa* Duch, “Benihoppe”, “Honeoye” “Sweet Charlie”, “Albion”, and “Monterey”. All of the varieties examined showed a similar pattern in terms of TGM in relation to developmental stage, i.e., only after the LG stage could TGM be effectively conducted. Nevertheless, for different varieties, TGM efficiency was indeed different. Compared to the other examined varieties, the fruits of ‘Benihoppe’ have moderate hardness and crispness, which is conducive to the diffusion of *Agrobacterium* and the expression of genes. Additionally, when injected with high concentrations of *Agrobacterium*, “Benihoppe” fruits were not easier to decay. Among the varieties examined, we found that *Fragaria ananassa* Duch, “Benihoppe”, was the best variety in terms of its fruit texture for target genes to be manipulated. We compared “Benihoppe” with “Monterey”, a variety that is largely different from “Benihoppe” in terms of its characteristics of floral initiation and fruit quality. As shown in Fig. 7, both GFP accumulation (Fig. 7a) and gene expression were much lower in ‘Monterey’ than in ‘Benihoppe’.

### Adoption of “percentage difference of phenotype” to evaluate gene function in fruit ripening

Although gene expression can be manipulated by TGM in strawberry fruit, the manipulation of a gene’s expression may not always induce a phenotypic change even if the gene’s product plays a role in strawberry fruit ripening. To accurately and precisely evaluate gene function in strawberry fruit ripening, we developed a method named the percentage difference of phenotype (PDP).

To demonstrate the applicability of this method, we evaluated the function of *FaMYB10*, a gene that has been reported to play a major role in pigment accumulation during fruit ripening^26^. Briefly, we sorted fruits into pairs, and from each fruit pair, we randomly selected one individual fruit to be injected with *Agrobacterium* carrying *FaMYB10* (treatment), while the other was injected with *Agrobacterium* carrying the empty vector (control). For each fruit pair, the fruit that became red first was recorded as “1” and the other as “0”; if there was no difference in reddening timing between the two fruits, they were both recorded as “1.” After all the fruit pairs were recorded, we calculated the percentage of recorded “1” values among the total number of fruit pairs for both the treatment and control and designated the percentage difference between the treatment and control groups as the PDP value (Table 1).

**Fig. 8:**
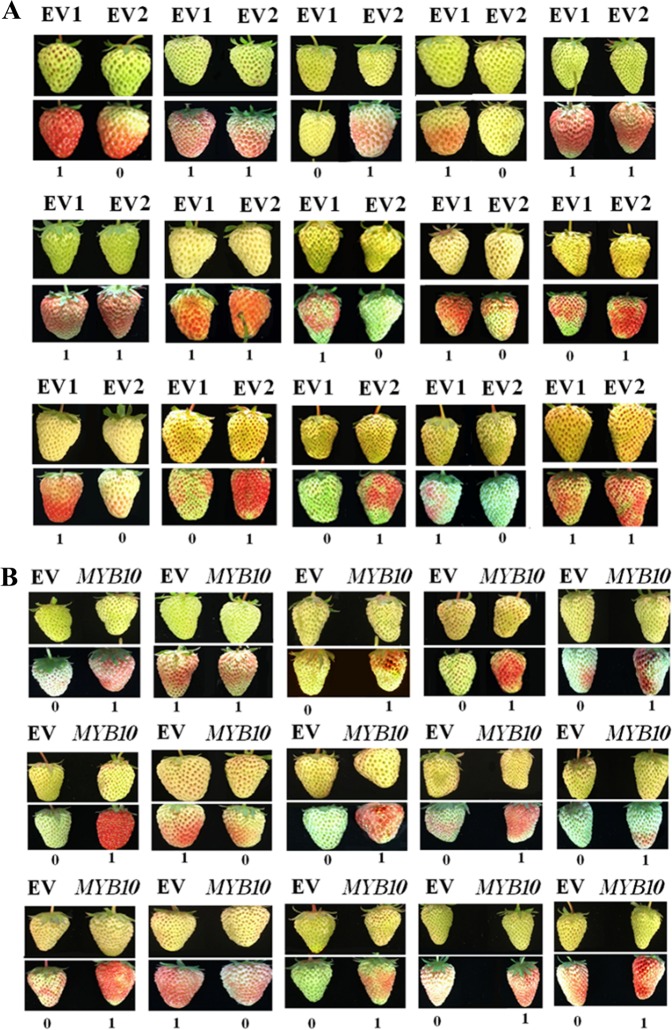
Analysis of PDP value and error for FaMYB10. Phenotype changes, showing the analysis of the PDP value and error according to the description in Materials and Methods (Table 1). **a** Control for PDP error analysis. Pairs of fruit (arbitrarily defined as EV1 and EV2) were infected with *Agrobacterium* carrying the empty vector. Fruit ripening status was determined based on coloration. The numbers “1” and “0” below the fruits refer to the fruit of the pair that ripened earlier (labeled “1”) and later (labeled “0”); if both fruits of a pair ripened simultaneously, they were both labeled “1.” **b** Assay of PDP value for FaMYB10. In each pair of fruit, one fruit was randomly selected and infected with *Agrobacterium* carrying FaMYB10, and the other was infected with *Agrobacterium* carrying the empty vector. The fruits were labeled as described in (**a**). For both (**a**) and (**b**), an assay with a batch of fruits is shown

**Table 1:** Calculation of the PDP value and error for *FaMYB10* based on the data provided in Fig. 8

Treatment	Number of “1” scores	Number of “0” scores	Percentage of “1” scores to total number of fruit pairs (%)	PDP value (%)	Notes
EV1	**11**	**4**	**73.33**	**13.33**	PDP error
EV2	**9**	**6**	**60.00**		
EV	**3**	**12**	**23.08**	**63.59**	PDP value for *FaMYB10*
FaMYB10	**13**	**2**	**86.67**		

As 15 pairs of fruit were used in this assay, the percentage of “1” and “0” scores refers to the ratios of the number of “1” or “0” scores to 15. The PDP error and value are the difference between EV1 and EV2 and between EV and *FaMYB10*, respectively

In theory, the ability of a gene to regulate fruit ripening should be positively correlated with its PDP value; i.e., a gene with no effect on fruit ripening should have a PDP value of 0%, and a gene whose effect on fruit ripening was sufficiently powerful should have a PDP of 100%. In practice, however, when working with a limited number of fruits, a PDP of 0% will seldom be obtained because the ripening process will not be completely identical between the two fruits of a pair. These innate differences in ripening would result in a spurious nonzero value for PDP, which could be interpreted as the PDP error. It is crucial to identify such errors to perform accurate gene functional identification.

To evaluate the PDP error, we replaced the vector carrying a target gene with the empty vector, i.e., we injected both fruits with empty vector. To distinguish between the two fruits, we randomly labeled them as EV1 (empty vector 1) and control EV2 (empty vector 2). The PDP value calculated from the paired fruits EV1 and EV2 represented the PDP error. Fig. 8a shows the phenotypic differences between the paired fruits induced by transformation with EV1 and EV2. Based on an analysis of the phenotypic changes, we calculated a PDP error of approximately 13.33% (Table 1). Figure 8b shows the phenotypic changes induced by *FaMYB10* transformation, from which a PDP value of approximately 63.59% was estimated (Table 1). This result indicates that the PDP method can be used to identify genes involved in regulating strawberry fruit ripening. Table 2 shows the PDP values and errors recorded for six experimental repeats. We calculated a *P*-value of 0.0003 by *t*-test analysis from these data, suggesting an absolute reliability of the PDP method in gene functional analysis.

**Table 2:** Statistical analysis of PDP value and error

Experimental repeat	1st	2nd	3rd	4th	5th	6th	mean	*t*-test between PDP value and error (*P*-value)
PDP value	**63.59**	**47.68**	**72.87**	**76.66**	**53.62**	**51.72**	**61.02**	**0.0003**
PDP error	**13.33**	**15.18**	**11.21**	**7.68**	**10.38**	**14.51**	**12.05**	

The PDP value and error assay was repeated six times. A *t*-test assay was carried out between the two sets of data obtained with experimental repeats. The *P*-value of 0.0003 implies that the difference between the PDP value and the error was extremely significant

Collectively, we optimized the traditional methods for transient gene manipulation and developed a fast, objective, and easy screen to effectively and precisely determine whether a gene regulates strawberry fruit ripening (Table 3).

**Table 3:** Summary of conditional optimization

Factor	Conditional optimization	Notes
•Vector type •Means of infection •Temperature •Time of infection •Developmental stage •Variety •TGM inconsistency	•VIGS vector is not recommended •Detached fruits, full injection •20~25 ℃ •4–6 days after initial infection •LG to W stage •*Fragaria ananassa* “Benihoppe” •Adoption of mixed fruit sample for measuring fruit-ripening-related parameters; adoption of PDP for gene function research	•Infection until whole fruit fully infiltrated •The most critical factor

## Discussion

### Limitations and recommendations on the use of TGM methods in strawberry fruits

A number of studies have reported TGM to be feasible for gene functional analysis in strawberry fruits^7,22–24,29–32^. However, our research raised some concerns about the use of TGM in gene functional studies. One major issue is that TGM is not powerful enough to effectively control strawberry fruit development and ripening, such that the observed effect of TGM is highly dependent on whether the treated fruit (i.e., the fruit subjected to target gene manipulation) is well matched with the control fruit (i.e., the fruit not subjected to gene manipulation). Alternatively, in some cases, TGM may not be capable of inducing the expected effect, even if the target gene indeed plays an important role in regulating strawberry fruit development and ripening.

In this study, we examined the factors that might affect the application of TGM to gene functional studies. Our results suggest that the limited effect of TGM may be ascribed to two major causes: the variation of TGM among individual fruits and the limitation of developmental stage. Even when there is consistency in both fruit developmental status and infection mode of *Agrobacterium*, eGFP images can vary greatly among different fruits; for instance, strong fluorescence was observed in some fruits, but weak or even no fluorescence was observed in others (Figs. 1 and 3). The reason for this variation is unknown.

The use of TGM in gene functional analyses of strawberry ripening has commonly been based on comparing fruit color between treated and control fruit, but the varied effect of TGM in individual fruits makes such comparisons complex, limiting the accuracy and reliability of the approach. An even more important concern is the limitation of fruit developmental stage. As shown in Fig. 6, the effect of TGM was tightly associated with fruit developmental stage: TGM induced *eGFP* gene expression and protein accumulation only at the LG and W stages, which implies that TGM could influence strawberry fruit development and ripening only after the LG stage. As the time interval from LG to FR was relatively short (10 days or so), and TGM started to work only 3 days after *Agrobacterium* infection, a TGM-induced change in the timing of fruit coloration could not be long-lasting, even if it was powerful enough to effectively control fruit development and ripening. This is an important limitation on TGM’s ability to substantially affect these processes.

### Necessity for exploiting PDP in gene functional studies

Because transient gene expression was reported to be feasible in strawberry fruits more than 10 years ago^7–9^, one might assume that it would be easy to perform gene functional studies in strawberry fruits. However, such studies are far more complex than expected. Gene functional identification has commonly been based on comparisons between the changes that take place during fruit coloration in treated and control fruits. However, problems arise from the limited effect of TGM, as well as the variation among individual fruits mentioned above. According to our studies, the maximum acceleration or delay in the fruit coloration process caused by TGM was commonly no more than 3–4 days. Unfortunately, no matter how carefully two fruits are matched, they may have innate differences in developmental processes, so there is no guarantee that the treatment and control fruits will redden on the same day. Although these innate differences are not very large (commonly 1–2 days), they may be large enough to obscure TGM-induced changes in the rate of fruit coloration. On the other hand, the variation of TGM among different fruits might further increase the difference between the treated and control fruits. These factors collectively make the exploitation of TGM in gene functional studies quite challenging. This problem can be effectively overcome by applying the PDP method to gene functional studies because this method is essentially based on a statistical analysis and hence allows conclusions to be drawn with more confidence. The applicability and reliability of PDP was thoroughly demonstrated in a functional analysis of *FaMYB10*, which is known to play a major role in pigment accumulation in strawberry fruits^26^, and the PDP method indeed yielded a PDP value of 63.59% for *FaMYB10*. However, the PDP value and error may vary between experiments due to differences between the batches of fruits used.

### Use of PDP for evaluating the relative ability of genes to regulate fruit ripening

Supposing that the paired fruits were both transformed with the control vector and that their ripening processes were absolutely identical, the PDP value obtained would be 0%. Likewise, if a gene was not at all involved in regulating fruit ripening, its PDP value would be 0%. In contrast, if a gene had a powerful effect on fruit ripening, its PDP value might reach 100%. However, owing to the innate variability of the rate of pigment accumulation between any two fruits (e.g., a treatment and control fruit pair), a zero value will seldom be obtained for a limited number of fruit pairs. However, although *FaMYB10* has been reported to be a critical regulator of pigment accumulation, its PDP value was only approximately 60%, which appears to imply that a 100% PDP value also cannot be obtained. Thus, a gene’s ability to control strawberry fruit ripening can be quantitatively reflected in its PDP value. This means that the PDP method can be used to compare the relative ability of different genes to regulate strawberry fruit ripening, which could be very useful in cases where researchers may be interested not only in knowing whether a single gene is implicated in fruit ripening but also in knowing what the relative effects of a given set of genes are in this regard. For example, the ABA receptor FaPYL1 has been reported to be involved in regulating strawberry fruit ripening^20^, but the ABA receptor family contains nine members; therefore, it would be useful to establish to what degree each individual member contributes to this effect. However, to establish the PDP analysis system for the first time, it is necessary to use *eGFP*, *GUS* or *LUC* as the selection marker gene and to detect the expression of detected genes with qRT-PCR to evaluate the error value and effects of transient expression.

### TGM is particularly feasible for studies of strawberry fruit ripening

Since TGM was reported more than 10 years ago^7^, it has commonly been thought that this technique could be exploited for molecular studies of strawberry fruit development and ripening. This study shows that TGM is feasible at the later stages of fruit development and ripening, beginning with the LG stage but not at MG or earlier stages (Fig. 6). This implies that the TGM technique may not be applicable to studying early fruit developmental stages. Fruit ripening is a complex process, and the transition from the large green stage to the white stage is known to be the key event in the initiation of fruit ripening^5,33^. Under our experimental conditions, the process from the white stage to the fully reddened stage normally took less than 10 days. Strikingly, the accumulation of eGFP protein was found to start on the third day and continue until the 10th day, in association with the process of strawberry fruit ripening (Fig. 5). These results suggest that TGM would be particularly suitable for molecular studies of strawberry fruit ripening.

TGM has normally been carried out in intact fruits, i.e., fruits attached to plants^7^. In this study, we compared the effect of TGM conducted in attached and detached fruits (Fig. 3). We speculate that different effects of TGM in the attached fruits might be due to changes in environmental factors, and the environmental temperature was found to be a critical factor determining TGM success. As the PDP process is largely dependent on fruit pairing, differences within pairs of fruits make it difficult to use PDP in a gene functional study. In comparison with attached fruits, studies using detached fruits have many advantages. These include easier control of experimental conditions, ease of pairing fruits, and increased effectiveness of TGM. This study demonstrates that PDP with detached fruits could be used for molecular studies of fruit ripening.

### Examination of protein accumulation and gene expression in relation to the evaluation of TGM

The use of TGM for gene functional studies is based on its control of the target gene’s expression. To demonstrate the effect of TGM, past studies have normally described the changes in the level of gene transcription resulting from TGM^21–24,34,35^. As shown in Fig. 5, however, the *eGFP* transcript could be detected immediately after *Agrobacterium* infection (i.e., on day 0), whereas eGFP protein started to be detected only at 3 days after *Agrobacterium* infection, suggesting that the altered pattern of gene transcription was not completely identical with the altered pattern of protein abundance. Since it is not possible for a gene to be integrated into the plant genome immediately after *Agrobacterium* infection, the transcripts detected immediately after the infection should be ascribed to production by *Agrobacterium*, not to the plant itself. This is not surprising, given that the fruits were injected with copious amounts of *Agrobacterium*. The time course of gene expression was basically identical to that of the protein; both *eGFP* gene expression and eGFP protein accumulation peaked on approximately the 4th day after *Agrobacterium* infection. Nonetheless, the effect of *Agrobacterium* on the level of target gene transcript needs to be addressed in experiments of this sort. Moreover, to show whether TGM is capable of effectively controlling a target gene’s expression, examining the protein level is recommended. As assessments of protein levels rely on the use of an antibody specific for that protein, when performing gene functional studies with TGM, it is beneficial to fuse the target gene to a tag whose corresponding antibody is commercially available.
